# Selective Cytotoxicity against Human Osteosarcoma Cells by a Novel Synthetic C-1 Analogue of 7-Deoxypancratistatin Is Potentiated by Curcumin

**DOI:** 10.1371/journal.pone.0028780

**Published:** 2011-12-21

**Authors:** Dennis Ma, Phillip Tremblay, Kevinjeet Mahngar, Jonathan Collins, Tomas Hudlicky, Siyaram Pandey

**Affiliations:** 1 Department of Chemistry and Biochemistry, University of Windsor, Windsor, Ontario, Canada; 2 Chemistry Department and Centre for Biotechnology, Brock University, St. Catharines, Ontario, Canada; Erlangen University, Germany

## Abstract

The natural compound pancratistatin (PST) is a non-genotoxic inducer of apoptosis in a variety of cancers. It exhibits cancer selectivity as non-cancerous cells are markedly less sensitive to PST. Nonetheless, PST is not readily synthesized and is present in very low quantities in its natural source to be applied clinically. We have previously synthesized and evaluated several synthetic analogues of 7-deoxypancratistatin, and found that JC-TH-acetate-4 (JCTH-4), a C-1 acetoxymethyl analogue, possessed similar apoptosis inducing activity compared to PST. In this study, notoriously chemoresistant osteosarcoma (OS) cells (Saos-2, U-2 OS) were substantially susceptible to JCTH-4-induced apoptosis through mitochondrial targeting; JCTH-4 induced collapse of mitochondrial membrane potential (MMP), increased reactive oxygen species (ROS) production in isolated mitochondria, and caused release of apoptosis inducing factor (AIF) and endonuclease G (EndoG) from isolated mitochondria. Furthermore, JCTH-4 selectively induced autophagy in OS cells. Additionally, we investigated the combinatory effect of JCTH-4 with the natural compound curcumin (CC), a compound found in turmeric spice, previously shown to possess antiproliferative properties. CC alone had no observable effect on Saos-2 and U-2 OS cells. However, when present with JCTH-4, CC was able to enhance the cytotoxicity of JCTH-4 selectively in OS cells. Such cytotoxicity by JCTH-4 alone and in combination with CC was not observed in normal human osteoblasts (HOb) and normal human fetal fibroblasts (NFF). Therefore, this report illustrates a new window in combination therapy, utilizing a novel synthetic analogue of PST with the natural compound CC, for the treatment of OS.

## Introduction

For many centuries, a plethora of natural products have been used in traditional medicine for the treatment of numerous ailments. One such product includes the *Curcuma longa* herb [1]. Traditionally, this herb has been used to treat anorexia, rheumatism, sinusitis, hepatic disorders, and inflammation [2]. More recently, a component of this herb, the compound curcumin (CC) also referred to as (1E,6E)-1,7-bis(4-hydroxy-3-methoxyphenyl)-1,6-heptadiene-3,5-dione or diferuloylmethane, has been recognized for its antiproliferative properties in treating cancer [1]. In particular, CC has been shown to regulate expression of genes implicated in cell proliferation, metastasis, chemotherapy resistance, and angiogenesis [1], [3]. The anti-neoplastic properties of CC are exhibited in many types of malignancies including breast cancer, colon cancer, kidney cancer, leukemia, prostate cancer, melanoma, and osteosarcoma (OS) [1], [4].

OS, a primary malignant bone tumor, is an extremely aggressive form of cancer associated with poor prognosis [5]. It occurs most commonly in the developing bones of children and adolescents and is often accompanied by lung metastases and subsequent respiratory failure [6], [7]. Current treatment strategies include surgery and radiation therapy with adjuvant chemotherapy with agents such as doxorubicin, cisplatin, methotrexate, etoposide, and ifosfamide at high doses [6]. Despite the use of these chemotherapeutics, the 5-year survival rate for patients with metastatic OS is only 20% [6]. Furthermore, toxicity has been associated with the use of these drugs and chemoresistance frequently develops in this aggressive cancer; thus, more selective and effective chemotherapeutics are needed for OS [7]–[11].

Previously, we have shown the natural compound pancratistatin (PST), isolated from the *Hymenocallis littoralis* plant, to induce cytotoxicity in a number of malignant cell lines at concentrations below 1 µM and reduce the volume of human prostate and colon tumor xenografts [12]–[18]. Contrasting from many approved chemotherapeutics in use, non-cancerous cell lines are markedly less sensitive to PST [12]–[18]. PST however, is present in only parts per million quantities in its natural source and there have been many difficulties associated with its chemical synthesis; therefore, the major bottleneck of this compound has been its low availability for preclinical and clinical work. We have recently synthesized and screened 7-deoxypancratistatin derivatives and have identified a C-1 acetoxymethyl analogue, JC-TH-acetate-4 (JCTH-4), with comparable efficacy and specificity to PST against several cancer cell lines [19].

Evasion of apoptosis, or type I programmed cell death, as a result of abnormalities in pathways leading to apoptosis plays a major role in the development of cancer; therefore, much effort has been made to manipulate and restore apoptosis as a way to treat cancer [20], [21]. Apoptosis can be activated in response to a death ligand binding to its corresponding death receptor or in response an internal stress stimulus such as DNA damage [22]. In response to such internal stress, various proapoptotic proteins are upregulated and translocated to the mitochondria where they induce mitochondrial membrane permeabilization, collapse of mitochondrial membrane potential (MMP), and release of apoptogenic factors which subsequently execute apoptosis directly or indirectly [23]. Execution of this pathway yields nuclear and cellular condensation, membrane blebbing, and formation of apoptotic bodies which are phagocytosed by phagocytes or neighbouring cells [24]. As well as apoptosis, much focus has been put on the implications of other cell death pathways in cancer therapy [25], [26].

Autophagy has been recognized as a cellular pro-survival response to various forms of sublethal cellular stress such as DNA damage, deficiencies in growth factors and nutrients, protein aggregates, reactive oxygen species, hypoxia, pathogens, and defective organelles [25]. Once this pathway is activated, cytoplasmic material is sequestered by double-membraned vesicles known as autophagosomes. These autophagosomes fuse with lysosomes to form autolysosomes which degrade the contained cytoplasmic contents [26]. Extensive activation of autophagy however, can result in a pro-death response leading to autophagic cell death, classified as type II programmed cell death [27].

In this report, we demonstrate JCTH-4 to be a potent inducer of apoptosis and autophagy in OS cells (Saos-2, U-2 OS) via mitochondrial targeting. Furthermore, CC was able to potentiate the cytotoxic effects of JCTH-4 in Saos-2 and U-2 OS cells. The normal human fetal fibroblast (NFF) and normal human osteoblast (HOb) cell lines utilized in this study were drastically less sensitive to JCTH-4 alone and in combination with CC, presenting a potential therapeutic window for this aggressive malignancy.

## Materials and Methods

### Cell Culture

An OS cell line, Saos-2 (American Type Culture Collection, Cat. No. HTB-85, Manassas, VA, USA), was grown in McCoy's 5A Medium Modified (Sigma-Aldrich Canada, Mississauga, ON, Canada) supplemented with 15% (v/v) fetal bovine serum (FBS) standard (Thermo Scientific, Waltham, MA, USA) and 10 mg/mL gentamicin (Gibco BRL, VWR, Mississauga, ON, Canada). An additional OS cell line, U-2 OS (American Type Culture Collection, Cat. No. HTB-96, Manassas, VA, USA), was grown in McCoy's 5A Medium Modified (Sigma-Aldrich Canada, Mississauga, ON, Canada) supplemented with 10% (v/v) FBS standard (Thermo Scientific, Waltham, MA, USA) and 10 mg/mL gentamicin (Gibco BRL, VWR, Mississauga, ON, Canada). Apparently NFF cells (Coriell Institute for Medical Research, Cat. No. AG04431B, Camden, NJ, USA) were cultured in Dulbecco's Modified Eagle's Medium, High Glucose (Thermo Scientific, Waltham, MA, USA) supplemented with 15% (v/v) FBS standard (Thermo Scientific, Waltham, MA, USA) and 10 mg/mL gentamicin (Gibco BRL, VWR, Mississauga, ON, Canada). HOb cells (Cell Applications, Inc., Cat. No. 406-05a, San Diego, CA, USA) were cultured in Osteoblast Growth Medium (Cell Applications, Inc., Cat. No. 417-500, San Diego, CA, USA). All cells were grown at 37°C and 5% CO_2_.

### Cell Treatment

After culturing cells to 60–70% confluence, they were treated with CC (Sigma-Aldrich Canada, Cat. No. C7727, Mississauga, ON, Canada), tamoxifen (TAM) citrate salt (Sigma-Aldrich, Cat. No. T9262, Mississauga, ON, Canada), the broad spectrum caspase inhibitor Z-VAD-FMK (EMD Chemicals, Gibbstown, NJ, USA), and JCTH-4 (((1S, 2S, 3R, 4S, 4aR, 11bR)-2, 3, 4-Trihydroxy-6-oxo-1, 2, 3, 4, 4a, 5, 6, 11b-octahydro-[1], [3]dioxolo[4,5-j]phenanthridin-1-yl)methyl Acetate) at the indicated doses and durations. As per a previously published protocol, JCTH-4 was produced by chemoenzymatic synthesis from bromobenzene [19]. All stock solutions of drugs were made with dimethylsulfoxide (Me_2_SO).

### Nuclear Staining

Post treatment and incubation with the aforementioned drugs, Hoechst 33342 dye (Molecular Probes, Eugene, OR, USA) was used to stain the nuclei. Cells were incubated with 10 µM Hoechst 33342 dye for 5 minutes and images were taken at 400× magnification on a Leica DM IRB inverted fluorescence microscope (Wetzlar, Germany).

### Annexin V Binding Assay

The Annexin V binding assay was performed to verify the induction of apoptosis. Following drug treatment, cells were washed two times using phosphate buffer saline (PBS), resuspended in Annexin V binding buffer (10 mM HEPES, 10 mM NaOH, 140 mM NaCl, 1 mM CaCl_2_, pH 7.6), and incubated with Annexin V AlexaFluor-488 (1∶50) (Sigma-Aldrich Canada, Mississauga, ON, Canada) for 15 minutes. Micrographs were taken at 400× magnification on a Leica DM IRB inverted fluorescence microscope (Wetzlar, Germany).

### WST-1 Assay for Cell Viability

The WST-1 based colorimetric assay was performed according to the manufacturer's protocol (Roche Applied Science, Indianapolis, IN, USA) to quantify cell viability as a function of active cell metabolism. 96-well clear bottom tissue culture plates were seeded with approximately 6.0×10^3^ Saos-2 cells/well, 7.5×10^3^ U-2 OS cells/well, 5.0×10^3^ NFF cells/well, or 4.0×10^3^ HOb cells/well and treated with JCTH-4 and CC at the indicated concentrations and for the indicated durations. Following treatment, the WST-1 reagent, which is processed to formazan by cellular enzymes, was added to each well and incubated for 4 hours at 37°C. Absorbance readings were taken at 450 nm on a Wallac Victor^3^™ 1420 Multilabel Counter (PerkinElmer, Woodbridge, ON, Canada) to quantify the formazan product and were expressed as percentages of the solvent control groups (Me_2_SO).

### Tetramethylrhodamine Methyl Ester (TMRM) Staining

TMRM (Gibco BRL, VWR, Mississauga, ON, Canada) was used as an indicator for MMP. Cells were grown on coverslips and treated with the indicated concentrations of JCTH-4 and CC for the indicated durations. Subsequent to drug treatment, cells were incubated with 200 nM TMRM for 45 minutes at 37°C. Micrographs were taken at 400× magnification on a Leica DM IRB inverted fluorescence microscope (Wetzlar, Germany).

### Mitochondrial Isolation

Mitochondria were isolated from untreated Saos-2 and U-2 OS cells. These cells were washed two times with cold PBS, resuspended in hypotonic buffer (1 mM EDTA, 5 mM Tris–HCl, 210 mM mannitol, 70 mM sucrose, 10 µM Leu-pep, 10 µM Pep-A, and 100 µM PMSF), manually homogenized, and subsequently centrifuged at 600× g for 5 minutes at 4°C. The supernatant was centrifuged at 15,000× g for 15 minutes at 4°C. The resulting cytosolic supernatant was discarded and the mitochondrial pellet was resuspended in cold reaction buffer (2.5 mM malate, 10 mM succinate, 10 µM Leu-pep, 10 µM Pep-A, and 100 µM PMSF in PBS).

### Amplex Red Assay

Generation of reactive oxygen species (ROS) was quantified with Amplex Red (Molecular Probes, Eugene, OR, USA). Opaque 96-well plates were equally loaded with isolated mitochondria suspended in cold reaction buffer, with 20 µg of protein/well. The Bio-Rad protein assay (Bio-Rad Laboratories, Hercules, CA USA) was used for protein quantification as per manufacturer's protocol. Isolated mitochondria were incubated with the indicated concentrations of drugs, Amplex Red reagent at a final concentration of 50 µM, and horseradish peroxidase (Sigma-Aldrich Canada, Mississauga, ON, Canada) in the ratio of 6 U/200 µL. Paraquat (PQ) (Sigma-Aldrich Canada, Mississauga, ON, Canada) was used as a positive control at 250 µM. Fluorescence readings were acquired after 2 hours of incubation at Ex. 560 nm and Em. 590 nm on a spectrofluorometer (SpectraMax Gemini XS, Molecular Devices, Sunnyvale, CA, USA). Fluorescence readings were expressed as relative fluorescence units (RFU).

### Treatment of Isolated Mitochondria & Evaluation of Apoptogenic Factor Release

Mitochondria isolated from Saos-2 and U-2 OS cells were directly treated with JCTH-4 and CC at the indicated concentrations for 2 hours in cold reaction buffer (2.5 mM malate, 10 mM succinate, 10 µM Leu-pep, 10 µM Pep-A, and 100 µM PMSF in PBS). The control group was treated with solvent (Me_2_SO). After treatment, mitochondrial samples were vortexed and centrifuged at 15,000× g for 15 minutes at 4°C. The resultant supernatant and mitochondrial pellet (resuspended in cold reaction buffer) samples were subjected to western blot analyses to monitor for mitochondrial release or retention of apoptogenic factors.

### Cellular Lysate Preparation

Cells were treated for 72 hours with the indicated concentrations of JCTH-4, CC, and TAM, and subsequently homogenized manually in cold hypotonic buffer (10 mM Tris HCl at pH 7.2, 5 mM KCl, 1 mM MgCl_2_, 1 mM EGTA, 1% Triton-X-100; 10 µM Leu-pep, 10 µM Pep-A, and 100 µM PMSF). Cell lysates were stored at -20°C until use.

### Western Blot Analyses

SDS-PAGE was performed on the protein samples. Electrophoresed proteins were transferred to a nitrocellulose membrane. For 1 hour, membranes were blocked with a 5% w/v milk TBST (Tris-Buffered Saline with Tween-20) solution. The membranes were probed with various primary antibodies overnight at 4°C for microtubule-associated protein1 light chain 3 (LC3) raised in rabbit (1∶500) (Novus Biologicals, Cat. No. NB100-2220, Littleton, CO, USA), β-Actin raised in mouse (1∶1000) (Santa Cruz Biotechnology, Inc., Cat. No. sc-81178, Paso Robles, CA, USA), apoptosis inducing factor (AIF) raised in rabbit (1∶1000) (Abcam, Cat. No. ab1998, Cambridge, MA, USA), endonuclease G (EndoG) raised in rabbit (1∶1000) (Abcam, Cat. No. ab9647, Cambridge, MA, USA), and succinate dehydrogenase subunit A (SDHA) raised in mouse (1∶1000) (Santa Cruz Biotechnology, Cat. No. sc-59687, Paso Robles, CA, USA). Following primary antibody incubation, membranes were washed once for 15 minutes and twice for 5 minutes with TBST and were incubated with an anti-mouse (1∶2000) or an anti-rabbit (1∶2000) horseradish peroxidase-conjugated secondary antibody (Abcam, Cat. No. ab6728 & ab6802, Cambridge, MA, USA) for 1 hour at 25°C. After secondary antibody incubation, the membranes were washed three times for 5 minutes in TBST. Bands were visualized with enhanced chemiluminescence reagent (Sigma-Aldrich Canada, Cat. No. CPS160, Mississauga, ON, Canada) and densitometry analyses were performed using ImageJ software.

### Monodansylcadaverine (MDC) Staining

MDC (Sigma-Aldrich Canada, Mississauga, ON, Canada) was used to detect autophagic vacuoles. Cells were grown on coverslips and treated with TAM, JCTH-4, and CC at the indicated concentrations and for the indicated durations. After treatment, cells were incubated with 0.1 mM MDC for 15 minutes. Using a Leica DM IRB inverted fluorescence microscope (Wetzlar, Germany), micrographs were taken at 400× magnification.

### Propidium Iodide (PI) Staining

PI (Sigma-Aldrich Canada, Mississauga, ON, Canada) was used to detect cell lysis. Cells were incubated with 1 µg/mL PI for 10 minutes and images were taken at 400× magnification on a Leica DM IRB inverted fluorescence microscope (Wetzlar, Germany).

## Results

### JCTH-4 causes selective cytotoxicity in OS cells in a time and dose-dependent manner

**Figure 1:**
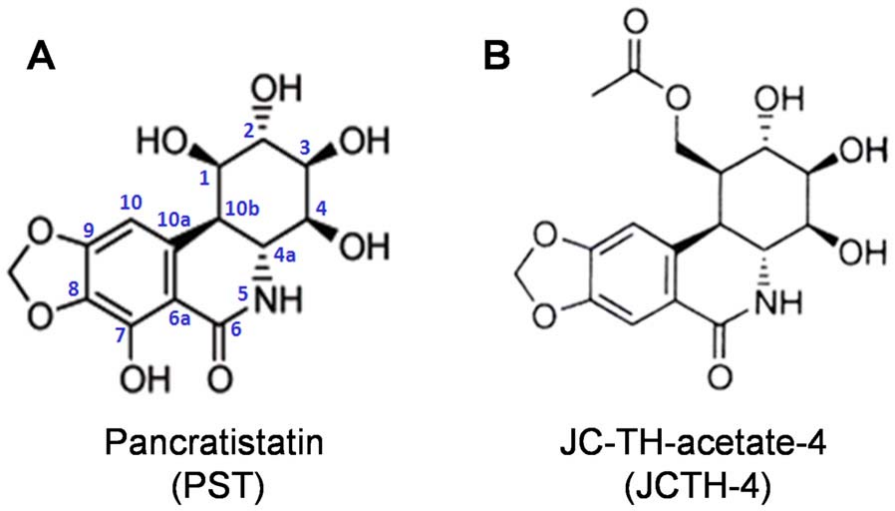
Comparison of chemical structures. Structures of (**A**) Pancratistatin (PST) and (**B**) JC-TH-acetate-4 (JCTH-4).

**Figure 2:**
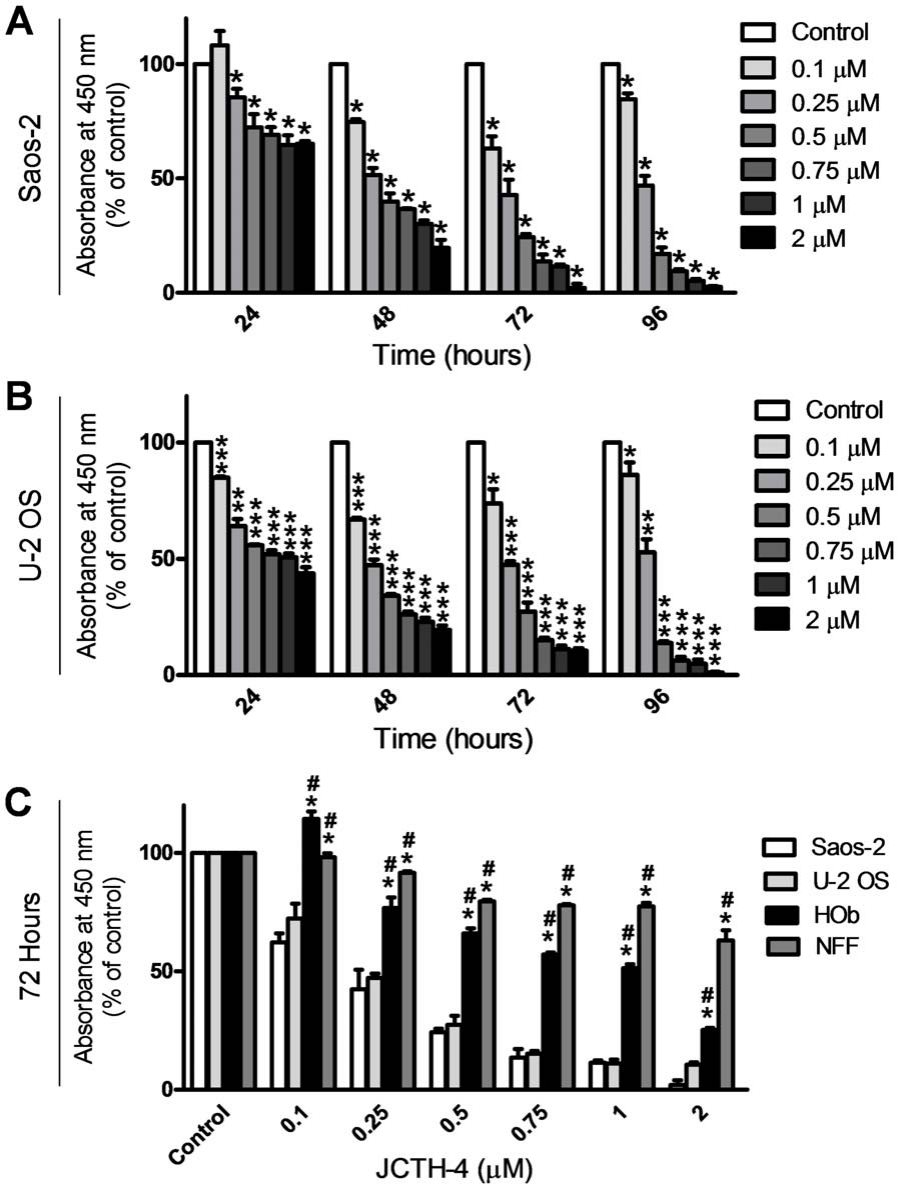
JCTH-4 causes selective cytotoxicity in OS cells in a time and dose-dependent manner. Effect of JCTH-4 on cellular viability of OS cells was determined by the WST-1 based colorimetric assay. (**A**) Saos-2 and (**B**) U-2 OS cells were treated with JCTH-4 and the WST-1 reagent was used to quantify cell viability. Absorbance was read at 450 nm and expressed as a percent of the control (Me_2_SO). Values are expressed as mean ± SD from quadruplicates of 3 independent experiments. *p<0.05, **p<0.01, ***p<0.001 versus solvent control (Me_2_SO). (**C**) Effect on cellular viability of HOb and NFF cells treated with JCTH-4 compared to Saos-2 and U-2 OS cells after 72 hours. The WST-1 reagent was used to quantify cellular viability. Absorbance was read at 450 nm and expressed as a percent of the solvent control (Me_2_SO). Values are expressed as mean ± SD from quadruplicates of 3 independent experiments. *p<0.005 versus Saos-2 cells; #*p*<0.005 versus U-2 OS cells.

As we have previously found PST (Figure 1A) to be effective in producing cytotoxicity selectively in various cancer cell lines, we evaluated the activity of JCTH-4 (Figure 1B) in the OS cell lines Saos-2 and U-2 OS using the WST-1 based colorimetric assay for cell viability. JCTH-4 decreased cell viability in a time and dose dependent manner and had a half-maximal inhibitory concentration (IC_50_) value of 0.25 µM after 48 hours in both Saos-2 and U-2 OS cells (Figure 2A,B). Importantly, JCTH-4 was selective to OS cells as HOb and NFF cells were drastically less sensitive to this compound (Figure 2C). Although HOb cells are the non-cancerous counterpart to the OS cell lines used in this study, we also evaluated the toxicity of this compound on another non-transformed non-cancerous human fetal fibroblast cell line (NFF) to confirm the selective anti-cancer activity of this compound.

### CC potentiates the cytotoxicity of JCTH-4 selectively in OS cells

**Figure 3:**
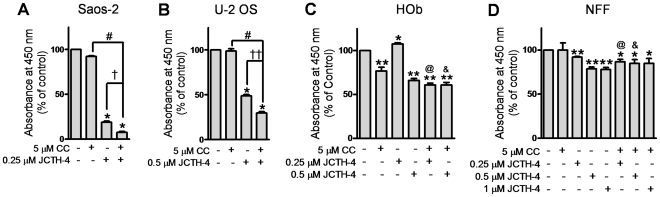
CC potentiates the cytotoxicity of JCTH-4 selectively in OS cells. Effect of JCTH-4 & CC in combination on cellular viability of OS cells was determined by the WST-1 based colorimetric assay. (**A**) Saos-2 (96 hours), (**B**) U-2 OS (72 hours), (**C**) HOb (72 hours), and (**D**) NFF (72 hours) cells were treated with JCTH-4 and CC and the WST-1 reagent was used to quantify cellular viability. Absorbance was read at 450 nm and expressed as a percent of the solvent control (Me_2_SO). Values are expressed as mean ± SD from quadruplicates of 3 independent experiments. *p<0.05, **p<0.01, versus solvent control (Me_2_SO); †*p*<0.001 versus 0.25 µM JCTH-4; ††*p*<0.01 versus 0.5 µM JCTH-4; #*p*<0.001 versus 5 µM CC; @*p*<0.001 versus 0.25 µM JCTH-4+5 µM CC treatment with Saos-2 cells (Figure 3A); &*p*<0.01 versus 0.5 µM JCTH-4+5 µM CC treatment with U-2 OS cells (Figure 3B).

The natural compound CC has been shown to possess anti-cancer properties as well as the ability to enhance the efficacy of various chemotherapeutics [28], [29]. Thus, we evaluated the combinatorial effects of CC and JCTH-4 in OS cells. CC alone at 5 µM had no significant effect on Saos-2 (96 hours) and U-2 OS cells (72 hours); however, when used in combination, CC was able to selectively potentiate the effect of JCTH-4 in these OS cell lines as HOb and NFF cells were markedly less sensitive to this combinatorial insult (Figure 3A–D).

### JCTH-4 alone and in combination with CC induces apoptosis selectively in OS cells

**Figure 4:**
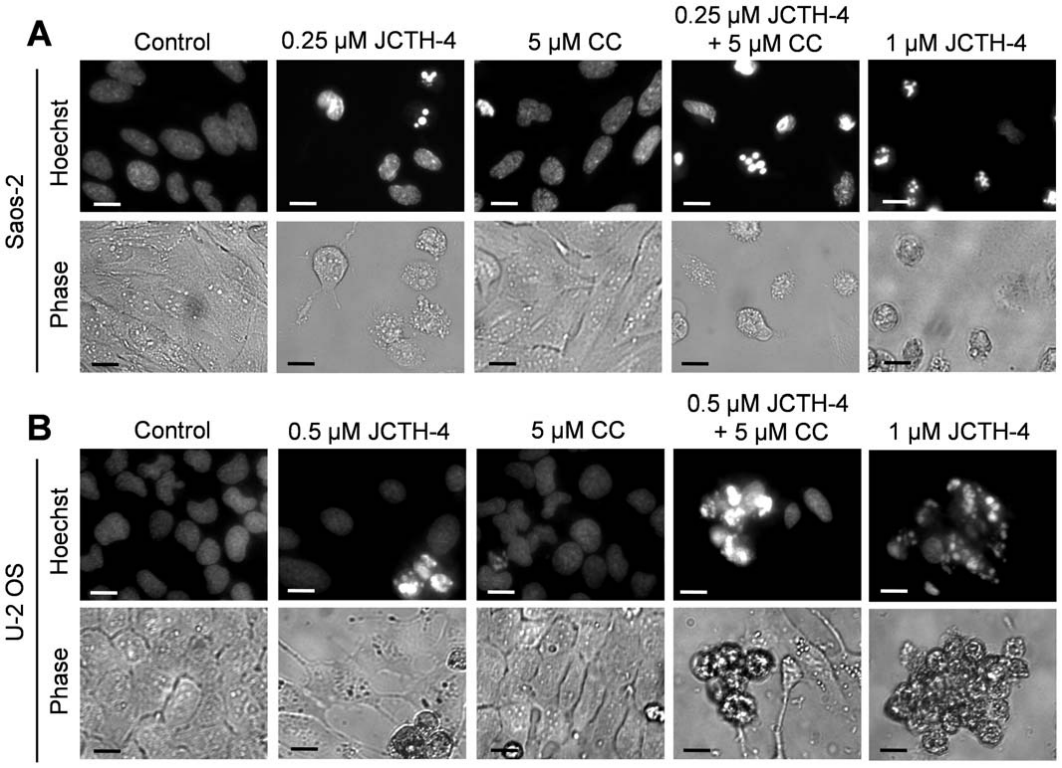
JCTH-4 alone and in combination with CC yields apoptotic morphology in OS cells. Nuclear and cellular morphology of (**A**) Saos-2 cells after 96 hours of treatment and (**B**) U-2 OS cells after 72 hours of treatment. Cells were treated with JCTH-4, CC, and solvent control (Me_2_SO). Post treatment, the cells were stained with Hoechst 33342 dye. Corresponding phase micrographs are shown below the Hoechst micrographs. Apoptotic morphology is evident in cells with bright and condensed nuclei accompanied by apoptotic bodies, as well as cell shrinkage and blebbing. Images were taken at 400× magnification on a fluorescent microscope. Scale bar = 15 µm. All images are representative of 3 independent experiments.

**Figure 5 pone-0028780-g005:**
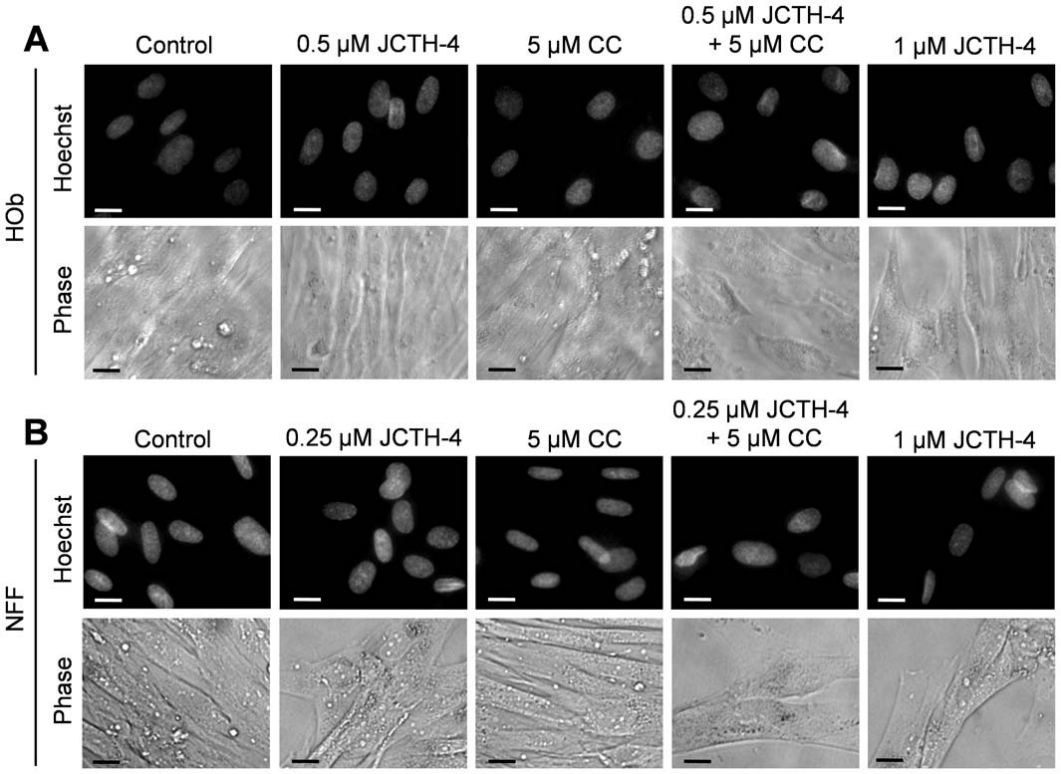
JCTH-4 and CC do not yield apoptotic morphology in HOb and NFF cells. Nuclear and cellular morphology of (**A**) HOb and (**B**) NFF cells after 72 hours of treatment. Cells were treated with JCTH-4, CC, and solvent control (Me_2_SO). Post treatment, the cells were stained with Hoechst 33342 dye. Corresponding phase micrographs are shown below the Hoechst micrographs. Apoptotic morphology is evident in cells with bright and condensed nuclei accompanied by apoptotic bodies, as well as cell shrinkage and blebbing. Images were taken at 400× magnification on a fluorescent microscope. Scale bar = 15 µm. All images are representative of 3 independent experiments.

**Figure 6 pone-0028780-g006:**
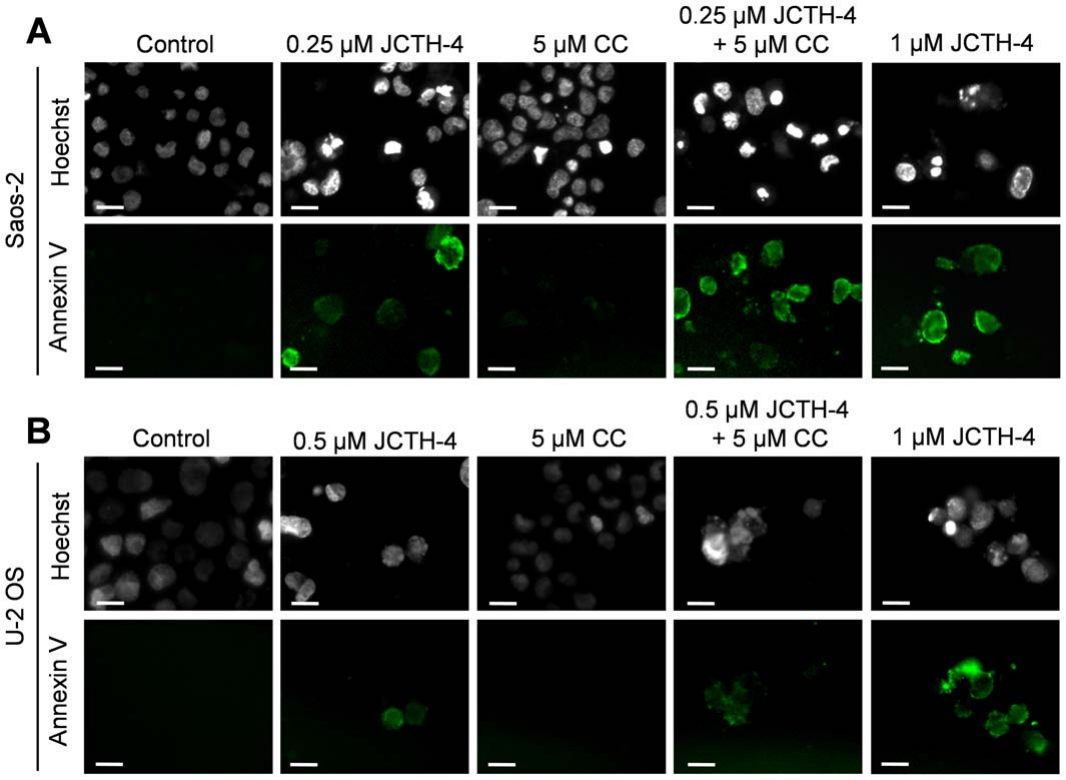
JCTH-4 alone and in combination with CC induces phosphatidylserine externalization in OS cells. Annexin V binding to externalized phosphatidylserine was monitored to confirm induction of apoptosis in (**A**) Saos-2 cells after 96 hours of treatment and (**B**) U-2 OS cells after 72 hours of treatment with the indicated concentrations of JCTH-4, CC, and solvent control (Me_2_SO). Cells were also stained with Hoechst dye. Images were taken at 400× magnification on a fluorescent microscope. Green fluorescence is indicative of Annexin V binding to externalized phosphatidylserine of the plasma membrane. Scale bar = 15 µm. All images are representative of 3 independent experiments.

**Figure 7:**
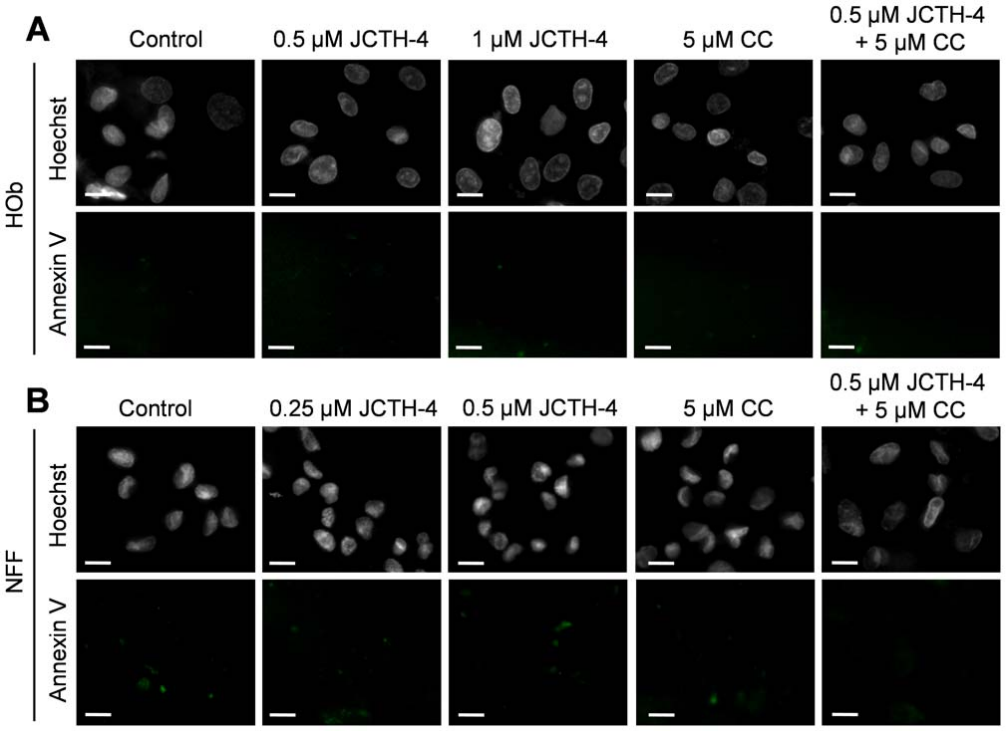
JCTH-4 alone and with CC does not induce phosphatidylserine externalization in HOb and NFF cells. Annexin V binding to externalized phosphatidylserine was monitored to confirm that apoptosis was not induced in (**A**) HOb and (**B**) NFF cells after 72 hours of treatment. Cells were treated with the indicated concentrations of JCTH-4, CC and solvent control (Me_2_SO). Cells were also stained with Hoechst dye. Images were taken at 400× magnification on a fluorescent microscope. Green fluorescence is indicative of Annexin V binding to externalized phosphatidylserine of the plasma membrane. Scale bar = 15 µm. All images are representative of 3 independent experiments.

Nuclear and cellular morphology was monitored in Saos-2 and U-2 OS cells treated with JCTH-4 and CC at the indicated concentrations for 96 and 72 hours respectively. As depicted by Hoechst staining, JCTH-4 produced brightly stained, condensed nuclei, and apoptotic bodies while corresponding phase micrographs revealed cell condensation and blebbing in a dose dependent manner; such features are indicative of apoptosis (Figure 4A,B). This morphology was not present in OS cells treated with 5 µM CC alone (Figure 4A,B); however, the frequency of these morphological changes induced by JCTH-4 was enhanced when used with 5 µM CC (Figure 4A,B). All of the aforementioned treatments did not produce apoptotic morphology in both HOb and NFF cells (Figure 5A,B). Selective induction of apoptosis by JCTH-4 alone and in combination with CC in OS cells was confirmed by Annexin V binding to externalized phosphatidylserine, a marker for apoptosis, indicated by green fluorescence (Figure 6–7) [30].

### JCTH-4 alone and in combination with CC targets OS cell mitochondria

**Figure 8:**
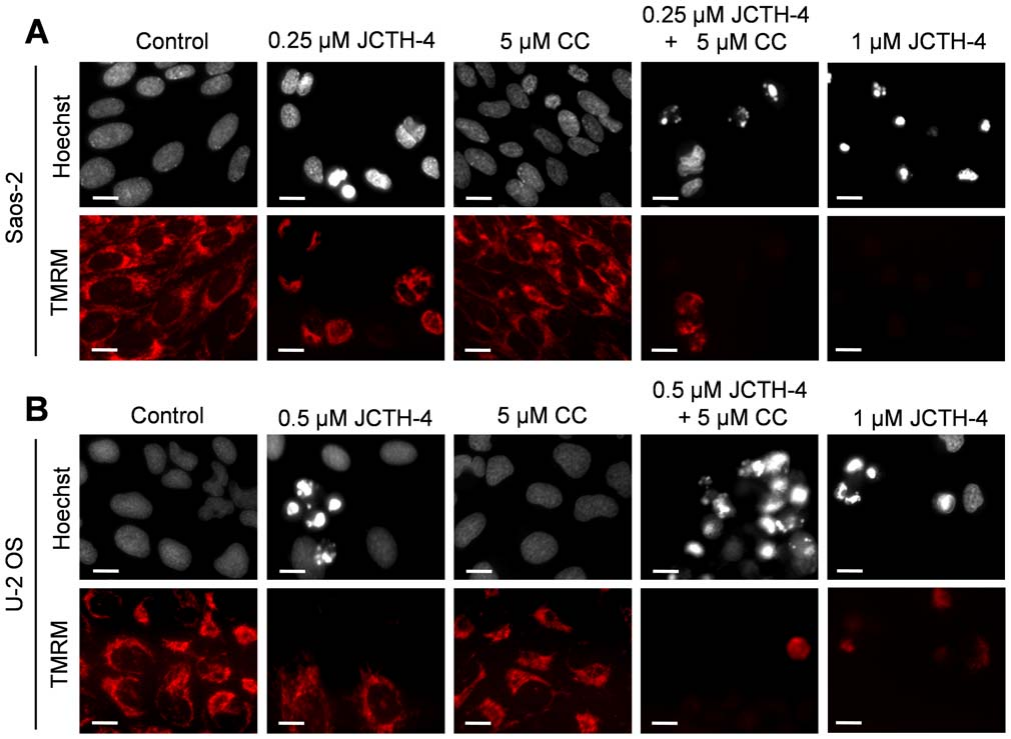
JCTH-4 dissipates MMP alone and in combination with CC in OS cells. Effect of JCTH-4 and CC on MMP in (**A**) Saos-2 cells after 96 hours of treatment and (**B**) U-2 OS cells after 72 hours of treatment was examined by TMRM staining. Cells were grown on coverslips, treated with the indicated concentrations of JCTH-4, CC, and solvent control (Me_2_SO) and stained with TMRM and Hoechst dye. Images were taken at 400× magnification on a fluorescent microscope. Red fluorescent punctuate marks are indicative of mitochondria with intact MMP. Scale bar = 15 µm. All images are representative of 3 independent experiments.

**Figure 9:**
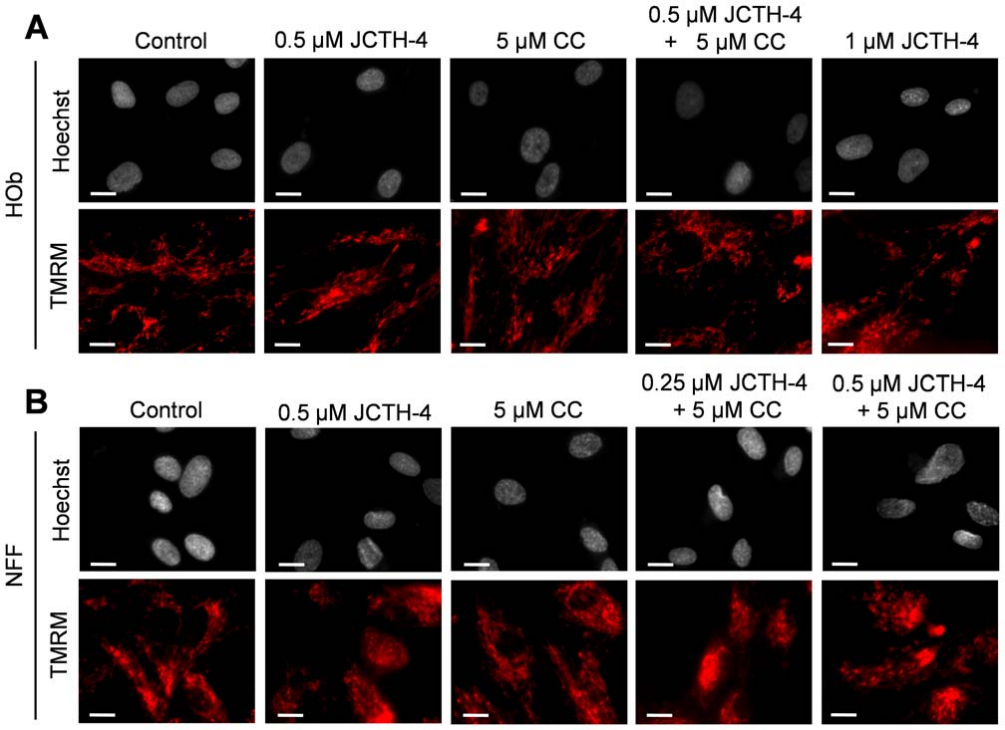
JCTH-4 and CC do not dissipate MMP in HOb and NFF cells. Effect of JCTH-4 and CC on MMP in (**A**) HOb and (**B**) NFF cells after 72 hours of treatment was examined by TMRM staining. Cells were grown on coverslips, treated with the indicated concentrations of JCTH-4, CC, and solvent control (Me_2_SO), and stained with TMRM and Hoechst dye. Images were taken at 400× magnification on a fluorescent microscope. Red fluorescent punctuate marks are indicative of mitochondria with intact MMP. Scale bar = 15 µm. All images are representative of 3 independent experiments.

In preceding studies, PST has been found to exert its effects by way of mitochondrial targeting; thus, mechanistic studies were performed to verify such targeting by JCTH-4 in OS cells [13]–[15], [16], [17]. Saos-2 and U-2 cells were treated for 96 and 72 hours respectively with JCTH-4 and CC at the indicated concentrations and stained with Hoechst dye and TMRM, an indicator of intact MMP and impermeabilized mitochondrial membrane, depicted by red fluorescence. Greatest dissipation of MMP was seen in OS cells treated with 1 µM JCTH-4 (Figure 8A,B). Dissipation of MMP was also seen with lower doses of JCTH-4; this dissipation by JCTH-4 was enhanced with the addition of 5 µM CC, which had no observable effect on MMP its own (Figure 8A,B). Additionally, none of these treatments yielded MMP dissipation in HOb and NFF cells (Figure 9A,B).

**Figure 10:**
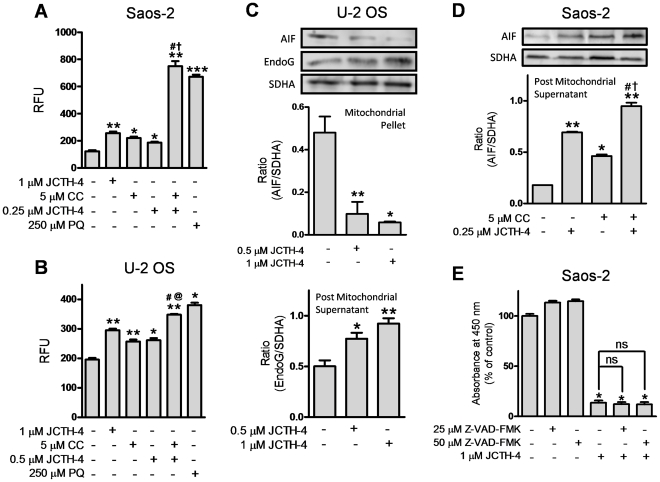
JCTH-4 directly causes mitochondrial ROS production and release of apoptogenic factors independent of caspases. (**A**) Saos-2 and (**B**) U-2 OS isolated mitochondria were treated directly with JCTH-4, CC, PQ, and solvent control (Me_2_SO), and incubated with Amplex Red and horseradish peroxidase for 2 hours. Subsequently, fluorescence readings were taken at Ex. 560 nm and Em. 590 nm and expressed as relative fluorescence units (RFU). Statistics were performed using GraphPad Prism version 5.0. Image is representative of 3 independent experiments demonstrating similar trends. Values are expressed as mean ± SD of quadruplicates of 1 independent experiment. *p<0.05, **p<0.01, ***p<0.001 versus solvent control (Me_2_SO); †*p*<0.01 versus 0.25 µM JCTH-4; @*p*<0.01 versus 0.5 µM JCTH-4; #*p*<0.01 versus 5 µM CC. Isolated mitochondria samples treated directly with JCTH-4, CC, and solvent control (Me_2_SO) for 2 hours were also centrifuged, producing mitochondrial pellets and post mitochondrial supernatants which were examined for retention and release of apoptogenic factors respectively via western blot analyses; (**C**) Retention of AIF and release of EndoG by U-2 OS cell mitochondria and (**D**) release of AIF by Saos-2 cell mitochondria was monitored. Mitochondrial pellets were probed for SDHA to serve as loading controls. Densitometric analyses were performed using ImageJ software and statistics were calculated using GraphPad Prism version 5.0. Image is representative of 3 independent experiments demonstrating similar trends. Values are expressed as mean ± SD of triplicates of one independent experiment. *p<0.01, **p<0.001 versus solvent control (Me_2_SO); †*p*<0.01 versus 0.25 µM JCTH-4; #*p*<0.01 versus 5 µM CC. (**E**) Saos-2 cells were treated with broad spectrum caspase inhibitor Z-VAD-FMK with and without JCTH-4 for 72 hours. WST-1 reagent was used to quantify cell viability. Absorbance was read at 450 nm and expressed as a percent of solvent control (Me_2_SO). Values are expressed as mean ± SD from quadruplicates of 3 independent experiments. *p<0.001 versus solvent control (Me_2_SO); ns = not significant.

Oxidative stress produced in the mitochondria has been associated the dysfunction of this organelle; in particular, mitochondrial permeabilization and apoptogenic factor release have both been linked to increases in ROS production [31]–[34]. After apoptogenic factors are released into the cytosol from the mitochondria, some can execute apoptosis by translocating to the nucleus or participating in cytosolic downstream pathways [23]. To evaluate the ability of JCTH-4 to induce the generation of ROS directly in mitochondria of OS cells, mitochondria were isolated from Saos-2 and U-2 OS cells, treated directly with various doses of JCTH-4 and CC for 2 hours, and analyzed for ROS production with Amplex Red. JCTH-4 alone at several concentrations caused an increase in ROS production in OS cells which was further enhanced by the addition of 5 µM CC (Figure 10A,B). 5 µM CC alone also increased ROS generation in isolated OS cell mitochondria (Figure 10A,B) PQ, known to cause ROS production in mitochondria, was used as a positive control [34]. Furthermore, isolated mitochondria of OS cells treated with JCTH-4 alone or with CC for 2 hours were monitored for the release of apoptogenic factors. Post treatment, mitochondrial samples were resuspended and centrifuged; resultant post mitochondrial supernatants and mitochondrial pellets were monitored by western blot analyses for the release and retention of apoptogenic factors respectively. By analyzing the resultant mitochondrial pellet samples of U-2 OS cells, isolated U-2 OS cell mitochondria treated with JCTH-4 retained less of the apoptogenic factor AIF (Figure 10C). JCTH-4 also caused the release of the apoptogenic factor EndoG in a dose dependent manner from these U-2 OS mitochondria, deduced by the analysis of the post mitochondrial supernatants (Figure 10C). Similarly, JCTH-4 and 5 µM CC alone caused the release of AIF from isolated mitochondria of Saos-2 cells; however, combinatorial treatment yielded the greatest release of AIF (Figure 10D).

The execution of apoptosis by released apoptogenic factors from the mitochondria can be achieved with the activation of proteins known as caspases, a family of cysteine proteases, or directly by the apoptogenic factor [23], [35]. To determine the dependence of caspases in JCTH-4-induced apoptosis, the broad spectrum caspase inhibitor Z-VAD-FMK was used at various doses in combination with JCTH-4. However, as determined through the WST-1 based colorimetric assay for cell viability, this inhibitor was not able to protect Saos-2 cells from JCTH-4 insult (Figure 10E). Thus, JCTH-4 exerts its cytotoxicity against OS cells in a manner independent of caspase activation.

### JCTH-4 selectively induces autophagy alone and with CC in OS cells

**Figure 11:**
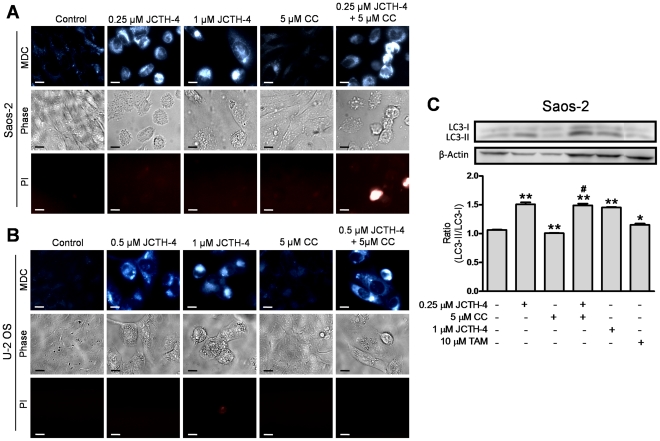
JCTH-4 induces autophagy in OS cells alone and with CC. The presence of autophagic vacuoles in (**A**) Saos-2 after 96 hours of treatment and (**B**) U-2 OS cells after 72 hours of treatment with JCTH-4, CC, and solvent control (Me_2_SO) was determined by MDC staining. Bright blue punctate marks are indicative of autophagic vacuoles. Corresponding phase and PI micrographs are shown below the MDC images. Scale bar = 15 µm. All images are representative of 3 independent experiments. (**C**) Cell lysates of Saos-2 cells treated with JCTH-4, CC, TAM as positive control, and solvent control (Me_2_SO) for 72 hours were examined for the conversion of LC3-I to LC3-II by western blot analyses. β-actin was probed to serve as a loading control. Densitometric analyses were done using ImageJ software and statistics were calculated using GraphPad Prism version 5.0. Image is representative of 3 independent experiments demonstrating similar trends. Values are expressed as mean ± SD of triplicates of one independent experiment. *p<0.05, **p<0.01 versus solvent control (Me_2_SO); #*p*<0.01 versus 5 µM CC.

Various studies have reported chemotherapeutics to trigger both pro-death and pro-survival autophagy [36]. Moreover, because JCTH-4 produced oxidative stress, a known autophagy inducer, we monitored OS cells for autophagic induction following treatment with JCTH-4 and CC. As illustrated MDC staining, OS cells treated with various doses of JCTH-4 produced blue punctate staining, indicative of autophagic vacuoles (Figure 11A,B). Such staining was also seen with the combinatorial treatment of 0.25 µM JCTH-4 and 5 µM CC, but not with 5 µM CC treatment alone (Figure 11A,B). Corresponding phase and PI images show cell shrinkage and blebbing with JCTH-4 treatment alone and in combination, but not with CC alone, while only combination treatment gave some positive PI staining (Figure 11A,B)

**Figure 12:**
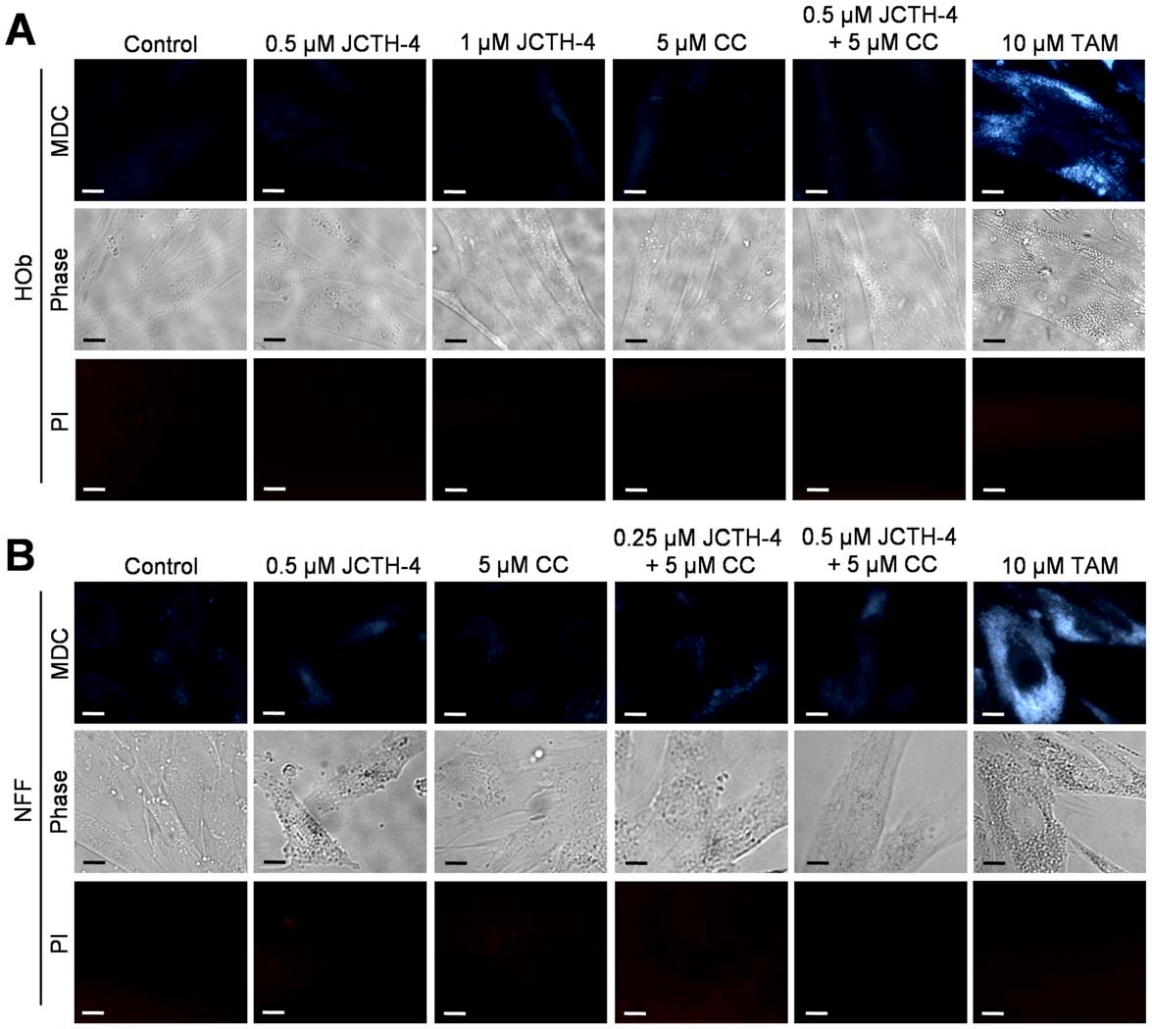
JCTH-4 and CC do not induce autophagy in Hob and NFF cells. MDC staining was used to detect the presence of autophagic vacuoles in (**A**) HOb and (**B**) NFF cells after 72 hours of treatment with JCTH-4, CC, and solvent control (Me_2_SO) at the indicated concentrations. Bright blue punctate marks are indicative of autophagic vacuoles. Corresponding phase and PI micrographs are shown below the MDC images. Scale bar = 15 µm. All images are representative of 3 independent experiments.

During autophagy, LC3 situated in the cytosol, referred to as LC3-I, is converted to LC3-II, a lipidated form of LC3 that is recruited to autophagosomal membranes [37]. To confirm the induction of autophagy in OS cells, western blot analyses were carried out on cell lysates of Saos-2 cells treated with the indicated concentrations of JCTH-4 and CC for 72 hours to monitor the conversion of LC3-I to LC3-II. TAM, a known inducer of autophagy, was used as a positive control. This conversion was enhanced with JCTH-4 at various concentrations (Figure 11C). With the treatment of 5 µM CC alone, LC3-II conversion decreased, and when used with 0.25 µM JCTH-4, the effect was not significantly different from 0.25 µM JCTH-4 alone (Figure 11C). In HOb and NFF cells, autophagic induction was only seen in the TAM positive control groups but not in cells treated with JCTH-4 and CC alone and in combination (Figure 12A,B). Therefore, JCTH-4 selectively induces autophagy in OS cells.

## Discussion

In this study, we demonstrate JCTH-4 to be a highly potent agent against OS cells. More specifically, JCTH-4 exerts its cytotoxicity against OS by way of apoptotic induction; OS cells treated with JCTH-4 exhibited cell shrinkage, blebbing, nuclear condensation, apoptotic body formation, phosphatidylserine externalization, and MMP dissipation (Figure 4,6,8). Moreover, by the end of 7 days of treatment, no viable cells were present, indicating the absence of any resistance to this compound by OS cells (data not shown). OS cells treated with JCTH-4 alone were negative for PI staining, a plasma membrane impermeable DNA stain, suggesting these cells were not dying by necrosis, a form of cell death characterized by lysis of the plasma membrane (Figure 11A,B) [38]. However, some PI positive staining was present in the combination treatment with CC, which could be a result of the permeabilization of cell membranes of late apoptotic cells (Figure 11A,B) [39]. Considerable toxicity to non-malignant tissue has been associated with a majority of the chemotherapeutics used for OS [40]–[44]. However, HOb cells, a normal non-cancerous equivalent to the OS cells used in this study, as wells as NFF cells exhibited markedly decreased sensitivity and did not show evident signs of apoptotic induction with JCTH-4 treatment (Figure 2C,5,7,9); therefore, JCTH-4 is selective towards OS cells and may prove to be a safer alternative for OS treatment to the current chemotherapeutics in use. Such selectivity suggests a specific target in cancer cells.

Previously, we have provided supporting evidence suggesting that PST targets cancer cell mitochondria [13], [17], [18]. Complimenting this data, we report similar results with JCTH-4. In addition to the JCTH-4-induced collapse of MMP in OS cells, observations made after direct JCTH-4-insult to isolated OS mitochondria strongly suggest this specific apoptotic activity to be attributed to mitochondrial targeting; with regards to isolated mitochondria from OS cells, JCTH-4 caused an increase in ROS generation and the release of apoptogenic factors (Figure 10). Likewise, PST selectively dissipated MMP with whole cell treatment and caused ROS production in isolated cancer cell mitochondria and not in non-cancerous cell mitochondria [13], [14]. The well known tumor suppressor p53 causes the expression of proapoptotic proteins that permeabilize the mitochondria in response to different forms of intracellular stress [45]. Our results proved JCTH-4-induced apoptosis to be p53 independent; Saos-2 cells, which do not express p53, and U-2 OS cells, with wild type p53, were equally sensitive to JCTH-4 insult (Figure 2A,B,4) [46], [47]. This circumvention in p53 activity strengthens the argument for a mitochondrial target as all p53 cellular events upstream of mitochondrial permeabilization have been proven to be insignificant in JCTH-4-induced cytotoxicity. As well as taking part in apoptosis pathways involved with the mitochondria, caspases are also utilized in apoptosis pathways independent of the mitochondria [35]. Akin to PST, we have found caspases to be nonessential in JCTH-4-induced apoptosis (Figure 10E), and that AIF and EndoG, two apoptogenic factors involved in caspase-independent pathways of apoptosis, are released from the mitochondria by direct JCTH-4 insult (Figure 10C,D); thus, caspase signalling upstream of mitochondrial membrane permeabilization and independent of mitochondria are not vital for the induction of apoptosis by JCTH-4, thereby further supporting the notion of a mitochondrial target by JCTH-4 [17], [23], [35], [48], [49]. It is this targeting of the mitochondria that may provide the means to cancer selectivity by JCTH-4.

Cancer cells possess many distinct characteristics, such as marked differences in their mitochondria and energy metabolism, which may be exploited for cancer selective therapy [50], [51]. Cancer cells possess a distinct metabolic phenotype, a phenomenon referred to as the Warburg effect, in which levels of aerobic glycolysis are elevated; thus, these cells have high glucose uptake and through the production of lactate, produce an acidic microenvironment [52]–[54]. Consequently, this glycolytic shift confers a proliferative advantage and an acquired resistance to apoptosis in cancer cells; intermediates of glycolysis provide a vast source of resources needed for nucleotide and lipid synthesis and alterations of the outer mitochondrial membrane have rendered it less susceptible to permeabilization [55]–[57]. Therefore, a promising strategy in cancer therapy would be to divert cancer cell metabolism away from glycolysis, which was demonstrated by other efforts via the promotion of oxidative phosphorylation [58]. Such a strategy may be employed by JCTH-4. A major factor promoting the resistance to apoptosis as a result of this metabolic shift is the glycolytic enzyme hexokinase; its association to the outer mitochondrial membrane has been shown to discourage mitochondrial membrane permeabilization [59], [60]. Cancer cells have also been reported to highly express antiapoptotic members of the Bcl-2 family of proteins and peripheral-type benzodiazepine, all of which inhibit mitochondrial outer membrane permeabilization [61], [62]. Thus, JCTH-4 may act by targeting these proteins.

Traditional medicines have long used natural products in treating various disorders. Modern science is now rediscovering many of these products and their potential in rectifying numerous pathological conditions [17], [63], [64]. The natural compound CC, used to treat an array of ailments, has been recognized to possess potent activity against numerous cancers, including OS [4], [65]. As determined by the WST-1 colorimetric assay, other reports have established IC_50_ values of 17.2 µM and 21.6 µM in Saos-2 and U-2 OS cells respectively after 72 hours [4]. By similar means, we found 5 µM CC to have very little to no effect on these cell lines (Figure 3A,B). However, when used with JCTH-4, CC was found to potentiate the cytotoxic effects of JCTH-4 in both Saos-2 and U-2 OS cells with HOb and NFF cells being markedly less sensitive to this combinatorial treatment (Figure 3A–D). Like JCTH-4 treatment alone, this combinatorial treatment exerts its cytotoxicity against OS cells selectively through induction of apoptosis (Figure 4–7). Although whole cell treatment of OS cells with 5 µM CC did not appear to effect viability of mitochondria (Figure 8), direct insult to isolated OS mitochondria induced ROS production and release of AIF (Figure 10A,B,D); such discrepancies between whole cell and direct mitochondrial treatment may be attributed to low solubility or uptake of CC [66]. In accordance with our findings, although at higher doses, other studies report whole cell treatment with CC to induce ROS production and AIF release from mitochondria [67], [68]. Interestingly, direct treatment of CC on OS mitochondria enhanced ROS production and AIF release (Figure 10A,B,D). CC is known to bind and target numerous proteins; however, these findings suggest CC to bind either one of these known targets and/or an unknown protein, both associated to the mitochondria [65], [69]. As JCTH-4 is shown to elicit its cancer selective effects via mitochondrial targeting, such targeting by CC may be responsible for sensitizing OS cells to JCTH-4-induced apoptosis.

Dual roles, both pro-death and pro-survival, have been implicated in autophagic induction elicited by chemotherapeutic insult [36]. JCTH-4 was found to induce autophagy at doses between 0.25 µM and 1 µM while 5 µM CC yielded no observable autophagic response in OS cells (Figure 11). The autophagic response produced by the combination treatment of 5 µM CC and 0.25 µM JCTH-4 was not significantly different from response made by the 0.25 µM JCTH-4 treatment alone; this may indicate the enhancement in activity of JCTH-4 by CC to be a result of targeting the apoptosis pathway rather than that of autophagy by CC. Previous work has shown CC alone to induce autophagy, although at much higher doses used in this study; inconsistencies between these findings and that of this report again may be attributed to the low solubility and uptake of CC [66], [70]. JCTH-4 treatment yielded autophagic induction which was accompanied by significant cytotoxicity and cell death, as depicted in the corresponding phase images and previous figures (Figure 2A,B, 3A,B, 4, 6, 11A,B); this may indicate JCTH-induced autophagy to be a detrimental response in OS cells. Such induction was not evident in HOb and NFF cells (Figure 12A,B); therefore, autophagic induction by JCTH-4 is selective towards OS cells. As established in this report, JCTH-4 causes mitochondrial ROS production and mitochondrial dysfunction. Oxidative stress is a known inducer of autophagy and thus, JCTH-4 may trigger autophagy as a default mechanism [25]. Regardless, because JCTH-4 permeabilizes OS cell mitochondria, these cells ultimately die from apoptosis.

Recapitulating the findings of this report, JCTH-4, was found to effectively induce apoptosis and autophagy in OS cells with evidence suggesting this to be a result of mitochondria targeting. Additionally, the natural compound CC was able to potentiate these cytotoxic effects induced by JCTH-4 against OS cells. Importantly, JCTH-4 treatment alone and in combination proved to be selective towards OS cells as non-cancerous cells were markedly less sensitive and did show evident signs of apoptotic and autophagic induction. Thus, we present a promising novel strategy, combining the novel synthetic compound JCTH-4 and the natural compound CC for the treatment of OS.
